# The Effect of Dietary Fish Oil in addition to Lifestyle Counselling on Lipid Oxidation and Body Composition in Slightly Overweight Teenage Boys

**DOI:** 10.1155/2011/348368

**Published:** 2011-07-09

**Authors:** Maiken Højgaard Pedersen, Christian Mølgaard, Lars Ingvar Hellgren, Jeppe Matthiessen, Jens Juul Holst, Lotte Lauritzen

**Affiliations:** ^1^Department of Human Nutrition, Faculty of Life Sciences, University of Copenhagen, 1958 Frederiksberg, Denmark; ^2^Department of Systems Biology, Technical University of Denmark, 2800 Lyngby, Denmark; ^3^Division of Nutrition, National Food Institute, Technical University of Denmark, 2860 Søborg, Denmark; ^4^Department of Biomedical Sciences, The Panum Institute, University of Copenhagen, 2200 København, Denmark

## Abstract

*Objective*. *n*-3 long-chain polyunsaturated fatty acids (LCPUFAs) have shown potential to increase lipid oxidation and prevent obesity. *Subjects*. Seventy-eight boys aged 13–15 y with whole-body fat% of 30 ± 9% were randomly assigned to consume bread with fish oil (FO) (1.5 g *n*-3 LCPUFA/d) or vegetable oil for 16 weeks. All boys were counselled to improve diet and exercise habits. *Results*. Lifestyle counselling resulted in decreased sugar intake but did not change the physical activity level. Whole-body fat% decreased 0.7 ± 2.5% and 0.6 ± 2.2%, resting metabolic rate after the intervention was 7150 ± 1134 kJ/d versus 7150 ± 1042 kJ/d, and the respiratory quotient was 0.89 ± 0.05 versus 0.88 ± 0.05, in the FO and control group, respectively. No group differences were significant. *Conclusion*. FO-supplementation to slightly overweight teenage boys did not result in beneficial effects on RMR, lipid oxidation, or body composition.

## 1. Introduction

Results from rodent studies have showed that dietary *n*-3 long-chain polyunsaturated fatty acids (LCPUFAs) increase fat oxidation and prevent high fat and sugar-induced obesity [1, 2]. Human studies have indicated similar effects of fish oil. A study by Couet et al. with healthy adults [3] and a study by Kabir et al. with diabetic women [4] both showed reduced body fat mass after fish oil interventions that lasted 3 weeks and 2 month, respectively. Hill et al., however, found no effect of fish oil on fat mass in a group of overweight subjects [5], but they and others [5–7] found that inclusion of fish oil in a hypocaloric diet increased weight loss.

Nakatani et al. found that fish oil supplementation increased the expression of uncoupling protein 2 (UCP-2) in rodent hepatocytes, which could explain the observed protection from weight gain [8]. Tsuboyama-Kasaoka et al. later used a UCP-2 knock out model to show that dietary fish oil prevents weight gain in rodents even in the absence of UCP-2, suggesting more than one pathway in mediating the effects of fish oil on weight control [9]. *n*-3 LCPUFAs have been shown to increase adiponectin concentrations in rodents and obese humans [10], and as adiponectin has been shown to increase fatty acid oxidation [11], this could also be a mechanism for the alleged antiadiposity effects of *n*-3 LCPUFAs. 

Considering the association between childhood obesity and later lifestyle diseases [12], early prevention of overweight and its sequelae is important. Studies on how children and adolescents respond to dietary interventions are few, and since it is problematic to extrapolate the results from interventions with adult subjects, data from intervention-studies in children are much needed. Therefore, we have investigated if *n*-3 LCPUFAs increase lipid oxidation, and improve body composition in slightly overweight teenage boys during their pubertal growth spurt.

## 2. Materials and Methods

### 2.1. Study Population

The study protocol was approved by The Committees on Biomedical Research Ethics of the Capitol Region of Denmark (H-A-2007-0055) and registered in the Clinical Trials database (http://www.clinicaltrials.gov/ no.NCT00929552). The recruitment and inclusion criteria have been previously described [13]. Our inclusion criteria were body mass for height above the 90th percentile and the average BMI of the included boys was comparable to 25 kg/m^2^ for adults [14].

### 2.2. Study Design and Intervention

The participants were randomly assigned to dietary supplementation with fish oil (FO) or a control oil blend for a 16-week intervention. The oils were included in bread, and the participants were asked to consume two pieces of rye bread and one wheat roll pr day. The intervention bread was produced for this study by Kohberg A/S (Bolderslev, Denmark). The fish oil was microencapsulated tuna oil from NuMega (Clover Corporation, Sydney, NSW, Australia), and the control oil was a 6 : 1 : 1 mix of palm shortening, soy oil, and rapeseed oil. We designed the study to provide the FO-group with 1.5 g/d LCPUFAs, which was the dose shown by Couet et al. to increase fat oxidation in adults [3]. However, random sampling of the bread revealed that the fat content was lower, providing the FO-group 4.9 (4.1–5.4) g/d (0.2 g/d eicosapentaenoic acid (EPA) + 0.9 g/d docosahexaenoic acid (DHA)) and 4.2 (3.0–5.0) g/d in the control group. (See full fatty acid composition in the bread in [13].) Both subjects and investigators were blinded to the treatment. Biomedical measurements were taken at baseline and after 16 weeks of intervention.

### 2.3. Life Style Intervention

The participants in this study were also subjected to a life style intervention, as we tried to educate and influence the boys towards healthier diets and a higher level of physical activity. Subjects in both groups received counselling by a dietician at the first examination visit and were further encouraged and supported in making healthy choices (regarding diet and exercise) every two weeks when they came in to pick up new supplies of bread. The initial counselling was based on a four-day food diary, including three weekdays and one weekend day, and results from a seven-day step recording with a pedometer (Yamax SW-200 Tokyo, Japan). The details about these recordings were published elsewhere [13].

### 2.4. Anthropometry, Body Composition, and Indirect Calorimetry

The boys were measured in the morning after an overnight fast. Height was measured to nearest cm, and weight to nearest 100 g on an electronic scale (Frederiksberg Vægtfabrik, Frederiksberg, Denmark). A measuring tape was used for waist and hip circumferences. Waist circumference was measured right below the ribs, and hip circumference was measured at the fullest part of the hip. Body composition was assessed by DXA scanning (Prodigy, General Electric Company, Madison, WI, USA). The scanner was calibrated each morning before use. Analysis of all scans was performed using enCore 2008 software (General Electric Company). 

Resting metabolic rate (RMR) and respiratory quotient (RQ) was measured by indirect calorimetry in a ventilated hood (Viasys Healthcare, Hoechberg, Germany). Metabolic testing was performed after 15 min supine of rest, and lasted for 25 min, of which the first 5 min were omitted from the later calculations. The calorimeters used room air for ventilation and for comparison with exhaust gas, and the analyzers were therefore calibrated to room air before each use and every 5 min during testing. Analyzers were furthermore calibrated to a standard gas sample (Air Liquide Danmark, Taastrup, Denmark) every day and tested every week with controlled alcohol burns. All readings were subsequently adjusted according to the results from the alcohol burn test.

### 2.5. Blood Sampling

Blood samples for the analysis of red blood cell (RBC) fatty acid composition, plasma adipokines and growth markers were drawn from the antecubital vein. The blood for RBC fatty acid analysis was collected in lithium-heparin-coated tubes, EDTA-coated tubes were used for analysis of leptin and adiponectin, and uncoated tubes for testosterone, insulin-like growth factor-1 (IGF-1), and insulin-like growth factor binding protein-3 (IGFBP-3). Blood samples were immediately placed on ice before centrifugation at 4°C with 2500 g for 10 min to isolate plasma, which was stored at −80°C until later analysis. RBCs were washed three times in isotonic saline and finally reconstituted 1 : 1 with isotonic saline with 0.005% butylated hydroxytoluene, and stored under nitrogen at −80°C.

### 2.6. Biochemical Analyses

Adiponectin was analyzed with a Milipore RIA kit (cat no. HADP-61HK, Billerica, MA, USA), and leptin was analyzed with a Human leptin RIA kit (cat no. HL-81K, Linco, St Charles, MO, USA). Interassay CV% was <9% for both adiponectin and leptin, and quality controls in both assays fell within expected limits. Testosterone, IGF-1, and IGFBP-3 concentrations were measured using an Immulite 1000 and kits from Siemens Medical Solutions Diagnostics (Los Angeles, CA, US). All CV% on the Immulite was <9%. The fatty acid composition in RBC was determined as previously described [14].

### 2.7. Statistical Analysis

Values are reported as mean ± SD if the data follow a normal distribution and as geometric mean (95% CI) if the data are not normally distributed. Potential effects of the intervention were analyzed using SAS 9.1 (SAS Institute Inc., Cary, NC, USA) with general linear models to adjust for the effect of covariates (ANCOVA). Baseline values of the outcome measure in question were included as covariate in all tests. Change in testosterone was furthermore included as covariate in the group comparisons of whole body fat percentage (fat-%), trunk fat-%, fat mass, hip and waist circumferences, and waist/hip ratio. The ANCOVA residuals were plotted to assess the need for transformation of the dependent variable. Within group changes from baseline to followup were assessed by paired *t*-tests. The study was based on power calculations to make it possible to show significant differences in the magnitude of 0.4 SD with *α* = 0.05 and *β* = 0.20 in a 2-sided test.

## 3. Results

The intervention was completed by 78 of the 87 teenage boys who originally entered the study. Six subjects dropped out from the FO-group and 3 from the control group. Reasons for withdrawal were: disliking the taste of the bread (5 from the FO-group and 2 from the control group), and insufficient structure in meal habits, which prevented them from eating the daily ration of bread (1 from each group). When asked after completion, 48% of the boys in the control group and 73% of the boys in the FO-group were able to guess which diet they had received. Compliance, estimated by asking the boys how much of the bread they had eaten during the intervention, was about 90% (70–100%) in both groups.

Baseline measurements showed that the boys were successfully randomized with respect to anthropometric measures and habitual diet (Table 1). During the 16-week intervention, the boys grew in height (1.8 ± 1.2 cm) and weight (2.0 ± 2.6 kg), and there was a significant increase in plasma testosterone of 0.6 ± 1.0 *μ*g/L. These developmental changes were similar for both groups. The lifestyle counselling resulted in a significant reduction in sugar intake and an increase in fibre intake for both groups. The boys in the FO-group significantly reduced fat intake compared with baseline, but the change was not different from that in the control group. No further differences were observed in the energy intake, the overall macronutrient composition of the diet, or the level of physical activity (steps per day).

The two groups had similar RBC fatty acid composition at baseline, but the RBC fatty acid composition at the end of the intervention period reflected that of the intervention oils (See [13]). In summary, DHA and EPA had increased by 78 and 100%, respectively, (DHA from 3.9 to 6.7 and EPA from 0.6 to 1.2% of total fatty acids) in the FO-group and by 14 and 20%, respectively, in the control group.

Whole body fat-% was significantly reduced in all boys after the intervention (*P* < 0.02), but no change was seen for the trunk fat-%. There was, however, no effect of fish oil on body composition compared with the control group (Table 2). No group differences were seen for hip and waist circumference, or the waist to hip ratio and RMR and RQ were also unaffected by the treatments.

**Figure 1:**
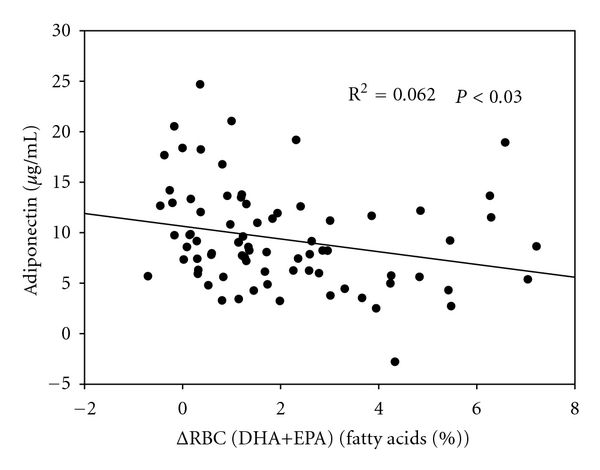
The correlation between the change in DHA+EPA content of RBC and plasma adiponectin at the end of the intervention adjusted for baseline values and changes in fat mass.

Leptin tended to decrease for the whole group (*P* = 0.06), and the change was best explained by the change in weight (*r* ^2^ = 0.138, *P* < 0.001), but the correlation with changes in fat mass was also significant (*r* ^2^ = 0.056, *P* < 0.04). Changes in adiponectin concentration correlated directly with the change in fat mass (*r* ^2^ = 0.061, *P* < 0.03). No group differences were found (Table 3), but there was a weak, but significant, inverse correlation between the change in the RBC-content of DHA+EPA and plasma concentrations of adiponectin at the end of the intervention adjusted for baseline values and changes in fat mass (Figure 1).

## 4. Discussion

In the present study, we did not find an effect of fish oil supplementation on metabolic rate and body composition in slightly overweight boys during puberty growth, nor did our results indicate any effect on fat oxidation as RQ was similar in the groups. These results on adolescent boys are in contradiction to the results from Couet et al., who found reduced RQ (suggesting increased lipid oxidation), and reduced fat mass in adults after only three weeks of fish oil intervention with doses only slightly higher than what we used [3], but in line with a recently published study from DeFina et al. who studied the effect of *n*-3 PUFA intake in combination with diet and excercise. In present study, the oil was a DHA-rich tuna oil, which resulted in a higher average DHA intake than what was attained by Couet and colleagues (0.9 versus 0.7 g/d). Thus, the lack of effect in present study cannot be explained by a too low DHA intake. It is also noteworthy that DeFina et al. used an EPA-rich oil at a higher level of intake than in our study (3 g/d of EPA+DHA) without observing effect on weight, metabolic rate, or RQ beyond those achieved with diet and exercise alone [16]. The weight reducing effect of fish oil has been consistent in rodent studies. However, most of the rodent studies have used high doses of fish oil. Nakatani et al. investigated the effects of different fish oil doses in female C57BL/6J mice and found that less than 40 E% fish oil was insufficient in reducing weight-gain [17]. Setting up a similar experiment to find the minimum dose for an effect on metabolism in humans would be highly interesting and may prove that the dose used in this study was too low for an effect to be detected. However, if the results parallel those in mice, that much fish oil is unrealistic in a human context, and using fish oil in weight management could be futile.

Because childhood obesity has long-lasting consequences, it is important to investigate potential health effects of commonly used foodstuff in children and adolescents. It is difficult to compare our results with studies in adults, as adolescents are not, metabolically, in a steady state, and changes in body composition in particular are controlled by mechanisms other than those seen in adults. Compared with the study of Couet et al. they had the advantage of a cross over design [3], which we were unable to match considering the unsteady state of our participants. The unsteady state might have increased the variation in the measurements due to differences in the pubertal development, which was a factor we tried adjusting for by including delta testosterone levels as covariate in most analyses. We, furthermore, studied a much larger group for a longer duration of time, which added to the quality of our study. It should be noted that the *n*-3 LCPUFAs in this study were included in bread and not given as pure oil. In one study the metabolic response to *n*-3 LCPUFAs was different when given as a salmon enriched diet compared with capsule supplementation, suggesting a possible matrix effect [18]. However, another study showed similar incorporation of *n*-3 LCPUFAs from capsules and fatty fish [19]. Unfortunately, we did not reach our intended dose of fish oil (1.5 g/d as in Couet et al. [3]), and we saw a slightly higher fat content of the bread in the FO-group, which may have counteracted a potential beneficial effect of the fish oil supplement. The difference in total fat intake caused by this might have been counter balanced somewhat as we also saw a significant reduction in dietary fat intake between baseline and follow up in the FO-group only. 

In our design, we tried to influence the participants to change lifestyle, which was included primarily for ethical reasons. The lifestyle intervention had no impact on the physical activity level, but sugar consumption had decreased for both groups, indicating that participation in the study had increased the boys' awareness of candy and soft drink usage. Fiber consumption was also increased during the intervention, but this is likely attributed to the fiber-rich bread provided by us, and not a lasting change in eating habits. The limited success of the lifestyle intervention shows that making a change in the most obvious bad habits is easier than making a commitment to increasing the physical activity level in spite of continuous encouragement to do so. The lifestyle intervention could have increased variation in the outcome measures or might have been a design advantage as previous studies have indicated that the effects of dietary fish oil was augmented when combined with increased exercise or reduced caloric intake [5–7]. There is also the risk that decreased sugar and increased fiber consumption had an impact on the outcome measures overriding that of the fish oil intervention.

Circulating leptin was reduced in a study with rats fed fish oil [20], but a human intervention study by Mori et al. showed no reduction after fish oil supplementation [21]. This agrees with our results as no group differences were found after 16 weeks of fish oil consumption. Leptin is a marker of adiposity [22], and plasma concentration of leptin has been shown to correlate with fat mass [22]. In our data, changes in leptin concentration correlated more strongly with changes in total body weight than with the changes in fat mass. *n*-3 LCPUFAs has been shown to increase adiponectin concentrations in rodents and obese humans [10]. In the present study, fish oil supplementation did not lead to an increase in adiponectin concentration, rather on the contrary, as we found a weak yet significant inverse association between the change in the RBC-content of EPA+DHA and changes in adiponectin concentration. Plasma concentrations of adiponectin have been shown to correlate inversely with fat mass [23], but in the present study changes in adiponectin concentration correlated directly with changes in fat mass. In a previous study in pubertal boys and girls, Schoppen et al. also did not find a significant correlation between plasma adiponectin and fat mass or fat%. However, they did find higher adiponectin levels in normal weight girls compared with overweight or obese girls, but this relationship was not seen for boys [24]. Thus, difference between our results and those from previous studies could be due to the male puberty, which was shown by Andersen et al. to affect adiponectin levels [25]. Girls experience metabolic and hormonal changes during adolescence that are very different from the male developments and might therefore respond differently to a similar treatment. The boys in this study were healthy and only mildly overweight, and it is not known if obese boys with more pronounced metabolic complications would respond differently to a fish oil intervention.

In conclusion, a 16-week fish oil intervention did not influence RMR or body composition in slightly overweight boys during their puberty growth spurt compared with the effect of a vegetable control oil. The antiobesity effect of *n*-3 LCPUFAs in adults has not been consistent, and more work is needed to investigate differences in the response to *n*-3 LCPUFAs in adolescents compared with adults.

##  Conflict of Interests

The authors declare no conflict of interests. 

## Figures and Tables

**Table 1:** Anthropometrics, habitual diet, and exercise^1^.

	Baseline		16 weeks		
	Control *n* = 40	Fish oil *n* = 38	Control *n* = 40	Fish oil *n* = 38	*P* ^2^
*Anthropometrics*					
Age (yrs)	14.3 ± 0.6	14.3 ± 0.7	14.6 ± 0.6	14.6 ± 0.7	
Weight (kg)	66.6 ± 9.9	69.8 ± 14.3	68.7 ± 10.3^∗3^	71.7 ± 14.4*	0.73
Height (cm)	169 ± 9	169 ± 11	171 ± 9*	170 ± 11*	0.45
BMI (kg/m^2^)^ 4^	23.1 (22.5–23.7)	24.1 (23.1–25.2)	23.3 (22.7–23.9)	24.3 (23.3–25.4)*	0.81
Testosterone (*μ*g/L)	3.0 ± 1.7	3.5 ± 1.7	3.6 ± 2.0*	4.0 ± 1.8*	0.48
IGF-1	360 ± 101	333 ± 93	352 ± 84	363 ± 86	0.19
IGFBP-3	5.5 ± 0.9	5.4 ± 0.6	5.4 ± 0.8	5.4 ± 0.6	0.08

*Habitual diet*					
Energy intake (kJ)	9292 ± 2523	8716 ± 2811	8524 ± 2258	8043 ± 2631	0.89
Carbohydrates (E%)	50.7 ± 5.1	51.3 ± 5.0	52.0 ± 6.7	52.7 ± 5.8	0.90
Protein (E%)	15.3 ± 2.9	15.5 ± 2.7	15.1 ± 2.9	16.2 ± 2.6	0.22
Fat (E%)^5^	33.8 ± 4.7	33.3 ± 5.3	32.9 ± 6.4	31.0 ± 5.7*	0.19
SFA (E%)	14.0 ± 2.4	14.0 ± 2.8	13.7 ± 3.3	12.8 ± 3.0*	0.21
MUFA (E%)	12.2 ± 2.2	11.6 ± 2.2	11.7 ± 2.6	10.9 ± 2.3*	0.56
PUFA (E%)	5.2 ± 1.2	5.2 ± 1.0	5.1 ± 1.3	4.9 ± 1.1	0.43
*n*-3 PUFA (E%)	0.3 ± 0.2	0.3 ± 0.2	0.3 ± 0.2	0.2 ± 0.2	0.51
Added sugar (E%)	10.8 ± 5.5	9.0 ± 5.0	8.6 ± 4.9*	6.7 ± 4.7*	0.98
Fibre (g/MJ)	1.9 ± 0.5	2.1 ± 0.6	2.2 ± 0.5*	2.5 ± 0.7*	0.16

*Physical activity*					
Steps pr day	10 950 ± 3842	10 711 ± 4253	11 901 ± 4234	11 134 ± 5395	0.43

^1^Data are presented as mean ± SD or geometric mean (95% CI).

^2^ *P* values for baseline adjusted group differences at 16 weeks.

^3∗^Indicates that the within-group-change from baseline to followup is significant (*P* < 0.05), assessed by paired *t*-tests.

^4^The usual cut-offs for BMI (>25 kg/m^2^ = overweight) is not applicable to children. The corresponding value for 13–15-year-old boys lies between 21.9 and 23.6 kg/m^2^ [15].

^5^This table compares habitual fat intake only, that is not including the contribution of energy and fat from supplemental oils.

**Table 2:** Metabolic rate and body composition^1^.

	Baseline		16 weeks		
	Control *n* = 40	Fish oil *n* = 38	Control *n* = 40	Fish oil *n* = 38	*P* ^2^
RMR (kJ/d)	6974 ± 913	7032 ± 1067	7150 ± 1134	7150 ± 1042	0.70^3^
RQ	0.88 ± 0.06	0.89 ± 0.07	0.89 ± 0.05	0.88 ± 0.05	0.62^3^
Whole body fat (%)	28.5 ± 8.8	31.1 ± 8.1	27.8 ± 8.8	30.5 ± 7.7	0.67
Trunk fat (%)	31.5 ± 9.5	31.7 ± 8.8	31.4 ± 9.2	31.1 ± 8.8	0.45
Fat mass (kg)	18.9 ± 6.2	21.9 ± 8.1	18.9 ± 6.1	22.1 ± 7.9	0.63
Hip circumference (cm)	94.9 ± 5.6	96.5 ± 8.3	95.9 ± 6.2	97.4 ± 8.7	0.94
Waist circumference (cm)	81.0 ± 6.4	83.1 ± 10.7	81.5 ± 6.0	83.0 ± 9.7	0.83
Waist/Hip	0.9 ± 0.1	0.9 ± 0.1	0.9 ± 0.1	0.9 ± 0.1	0.88

^1^Data are presented as mean ± SD.

^2^ *P* values describe group differences at 16 weeks adjusted for baseline and Δ testosterone.

^3^The analysis of RMR and RQ was only adjusted for baseline values.

**Table 3:** Plasma concentrations of adipokines^1^.

	Baseline		16 weeks		
	Control *n* = 40	Fish oil *n* = 38	Control *n* = 40	Fish oil *n* = 38	*P* ^2^
Leptin (ng/mL)	7.6 (5.8–9.9)	7.4 (5.7–9.7)	7.2 (5.6–9.2)	6.9 (6.8–9.9)	0.99
Adiponectin (*μ*g/mL)	8.7 (7.3–10.4)	8.0 (6.7–9.6)	7.9 (6.4–9.5)	8.2 (6.8–10.0)	0.49

^1^Data are presented as geometric mean (95% CI).

^2^ *P* values describe group differences at 16 weeks adjusted for baseline and Δ fat mass.
